# Muco-cutaneous manifestations in HIV infection/AIDS

**DOI:** 10.1186/1471-2334-14-S4-O14

**Published:** 2014-05-29

**Authors:** Vasile Benea, Simona Roxana Georgescu, Mircea Tampa, Mihaela Anca Benea, Elisabeta Otilia Benea, Șerban Benea

**Affiliations:** 1 Department of Dermatology, Clinical Hospital of Infectious and Tropical Diseases “Dr. Victor Babeş”, Bucharest, Romania; 2 National Institute for Infectious Diseases “Prof. Dr. Matei Balş”, Bucharest, Romania

## 

The HIV/AIDS infection is an important public health problem in Romania. The aim of this study was to evaluate the main muco-cutaneous manifestations and their frequency in patients with HIV infection/AIDS in Romania.

The study was performed beginning 1988 among 400 patients with HIV infection/AIDS who attended Scarlat Longhin Clinical Hospital for Dermatology, Victor Babeş Clinical Hospital of Infectious Diseases and Matei Balş National Institute for Infectious Diseases Bucharest, Romania. The patients were clinically examined; mycological examination (KOH exam, cultures) was necessary in 216 cases and histological examination was performed in 32 cases.

The subjects were classified in all stages of disease. The age of patients included in the study ranged from 6 months to 82 years. Muco-cutaneous manifestations were present in 76% of patients. The main muco-cutaneous manifestations registered were infections - 283 cases (oral and/or genital candidiasis - 179 cases; *molluscum contagiosum* - 53 cases; warts - 37 cases; tinea - 36 cases; sexually transmitted infections - 107 cases: herpes simplex genitalis - 33 cases, urethritis - 31 cases, *condylomata acuminata* - 29 cases, syphilis - 17 cases; herpes zoster - 28 cases; scabies - 12 cases; intertrigo - 11 cases; hairy leukoplakia - 4 cases; acute retroviral infection - 2 cases; disseminated cryptococcosis - 2 cases; bacillary angiomatosis, ganglionar tuberculosis - 1 case each), xerosis - 172 cases, papular eruptions - 87 cases, seborrheic dermatitis - 57 cases, atopic dermatitis - 24 cases, drug reactions - 21 cases, cancers - 20 cases (Kaposi sarcoma - 16 cases, basal cell carcinoma - 4 cases), psoriasis - 7 cases.

Muco-cutaneous manifestations in HIV infection/AIDS are frequent and polymorphous. They can occur at any point in time, but their frequency and severity are increasing as HIV infection progresses. Dermatological examination is an important step in evaluation of patients HIV-positive.

